# Letter to the Editor Regarding: “Postural Hypervigilance and Perception of Correct Sitting Posture in Individuals with and without Low Back Pain”

**DOI:** 10.1055/s-0044-1779334

**Published:** 2024-03-21

**Authors:** Giampierre Antonio Cortez Bocangel

**Affiliations:** 1 Universidad Ricardo Palma, Lima, Peru

Mr. editor,

I have read with great pleasure and gratitude the article published in the journal that you chair with DOI - https://doi.org/ 10.1055/S-0042-1756154 - “Postural Hypervigilance and Perception of Correct Sitting Posture in Individuals with and Without Low Back Pain” from published online: 2022–10–13 by Dr. Eduardo Lima de Oliveira. First of all, I would like to congratulate the authors for their rigorous work, since their methodology is solid and their results are clear and concise.

In recent years, low back pain or back pain has been the leading cause of disability worldwide, with a higher prevalence in the elderly, in the economically active population and in low- and middle-income countries. 1 Carrying out a study like this has important implications both for daily life, clinical practice, sports practices and also in the workplace, since up to 73% of those who suffer a first episode of pain will suffer a recurrence. 2 In Latin America, low back pain (LBP) related to work activities occurs in approximately one third of workers. 3 These findings suggest that Postural Hypervigilance (PH) may be a common factor in the perception of correct posture, regardless of whether or not a person experiences DL. In Peru, studies performed found that the prevalence of low back pain was 7.07%. 4

That is why it is crucial to investigate whether PH and the ability to perceive an adequate posture have some relationship in the appearance and/or maintenance of low back pain, as well as in the quality of life of the people who suffer from it; In this way, health professionals could develop treatment or prevention strategies to help patients create appropriate habits and thus avoid extreme or forced postures. In addition, by determining that PH is not related to low back pain, people could take other measures to improve their posture, promoting proper ergonomics and the adoption of adequate postures without falling into PH.

I hope that research like this one will continue and may even be expanded in the future to improve our understanding of how LP-related PH can affect people's quality of life, since the latter is a prevalent condition in the entire population and is the main cause of functional loss and work absenteeism. 5 Due to all of the above mentioned, it would also be convenient to look for associations between other types of back pain such as cervical pain or dorsal pain to have a broader picture.

## References

[1] Hartvigsen J Hancock M J Kongsted A Qué es el dolor lumbar y por qué debemos prestar atención Lanceta. 2018 391(10137):2356 2367

[2] Lazarte Argandoña G A Eslava Parra D B Prevalencia y factores asociados a la lumbalgia y discapacidad por dolor lumbar en vigilantes de Miraflores [tesis] Perú Universidad Peruana de Ciencias Aplicadas 2017(citado 5 de julio de 2020). Disponible en:https://repositorioacademico.upc.edu.pe/handle/10757/621858

[3] Donelson R McIntosh G Hall H Is it time to rethink the typical course of low back pain? PM R 2012 4 06 394 401, quiz 400 22381638 10.1016/j.pmrj.2011.10.015

[4] Gamboa R Medina M Acevedo E Prevalencia de enfermedades reumatológicas y discapacidad en una comunidad urbano-marginal: resultados del primer estudio COPCORD en el Perú Rev Per Reumatol 2009 15 01 40 46

[5] Popescu A Lee H Neck pain and lower back pain Med Clin North Am 2020 104 02 279 292 32035569 10.1016/j.mcna.2019.11.003

