# Supplementary material for: Letter to the Editor Regarding: “Postural Hypervigilance and Perception of Correct Sitting Posture in Individuals with and without Low Back Pain”
Source: Rev Bras Ortop (Sao Paulo). 2024 Mar 21;59(1):e148–9. doi: 10.1055/s-0044-1779334 (PMC10957281; doi:10.1055/s-0044-1779334)
Supplement: Related texts

# Research article: Carta ao Editor sobre: “Hipervigilância postural e percepção da postura correta sentada em indivíduos com e sem dor lombar”

Senhor Editor,

Li com muito prazer e gratidão o artigo publicado na revista que você preside junto ao DOI - https://doi.org/ 10.1055/S-0042-1756154 - “Hipervigilância Postural e Percepção da Postura Correta Sentada em Indivíduos com e sem Dor Lombar” publicado online: 2022–10–13 pelo Dr. Eduardo Lima de Oliveira. Em primeiro lugar, gostaria de felicitar os autores pelo seu trabalho rigoroso, pois a sua metodologia é sólida e os seus resultados são claros e concisos.

Nos últimos anos, a dor lombar ou dor nas costas tem sido a principal causa de incapacidade em todo o mundo, com maior prevalência nos idosos, na população economicamente ativa e em países de baixa e média renda. 1 A realização de um estudo como este tem implicações importantes tanto na vida cotidiana, na prática clínica, nas práticas desportivas e também no local de trabalho, uma vez que até 73% daqueles que sofrem um primeiro episódio de dor sofrerão uma recorrência. 2 Na América Latina, a dor lombar (lombalgia) relacionada às atividades laborais ocorre em aproximadamente um terço dos trabalhadores. 3 Esses achados sugerem que a Hipervigilância Postural (HP) pode ser um fator comum na percepção da postura correta, independentemente de a pessoa vivenciar ou não DL. No Peru, estudos realizados constataram que a prevalência de dor lombar foi de 7,07%. 4

Por isso é fundamental investigar se a HP e a capacidade de perceber uma postura adequada têm alguma relação com o surgimento e/ou manutenção da dor lombar, bem como com a qualidade de vida das pessoas que sofrem com isso. Dessa forma, os profissionais de saúde poderiam desenvolver estratégias de tratamento ou prevenção para ajudar os pacientes a criar hábitos apropriados e evitar posturas extremas ou forçadas. Além disso, ao determinar que a HP não está relacionado à dor lombar, as pessoas poderiam adotar outras medidas para melhorar sua postura, promovendo uma ergonomia adequada e a adoção de posturas corretas sem recorrer à HP.

Espero que pesquisas como esta continuem e possam até ser ampliadas no futuro para aprimorar nossa compreensão de como a HP relacionada à dor lombar pode afetar a qualidade de vida das pessoas, uma vez que esta é uma condição prevalente em toda a população e a principal causa de perda funcional e absenteísmo no trabalho. 5 Por tudo o que foi mencionado acima, também seria conveniente procurar associações entre outros tipos de dores nas costas, como dores cervicais ou dorsais, para ter um quadro mais amplo.
